# 

**Published:** 1894-01-06

**Authors:** 


*THE HOSPITAL.*

**PRICE TWOPENCE.**

**No. 380, Vol. XV.—Jan. 6th, 1894.**

[Registered at the General Post Office as a Newspaper for transmission in the United Kingdom and Abroad.]

JANUARY 6, 1894.

**WITH EXTRA NURSING SUPPLEMENT.**

**Per Annum, 8s. 6d., post free.**

**Monthly Parts, 6d.; post free, 8d.**

**Ditto per Annum, 8s., post free.**

### THE HOSPITAL

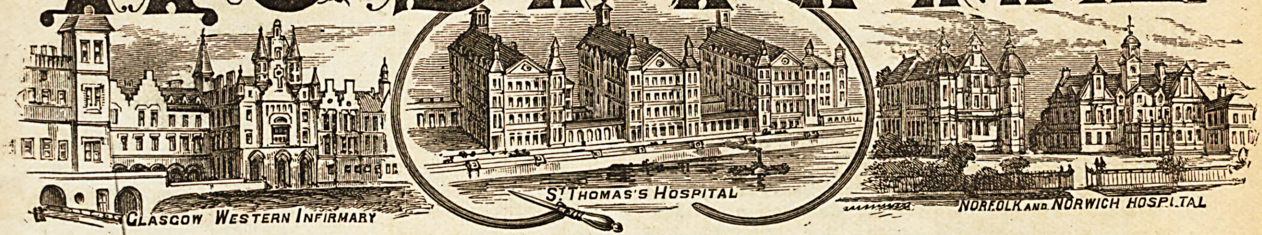

No. 380. Vol. XV. **WITH EXTRA NURSING SUPPLEMENT.** SATURDAY, JANUARY 6TH, 1894.

